# Improving Pharmacy Staff Knowledge and Practice on Childhood Diarrhea Management in Vietnam: Are Educational Interventions Effective?

**DOI:** 10.1371/journal.pone.0074882

**Published:** 2013-10-03

**Authors:** Duc Minh Pham, Mona Byrkit, Hoang Van Pham, Trung Pham, Chien Thang Nguyen

**Affiliations:** 1 PATH, Hanoi, Vietnam; 2 Institute of Social and Medical Studies, Hanoi, Vietnam; 3 Management Science for Health, Hanoi, Vietnam; Aga Khan University, Pakistan

## Abstract

**Background:**

In many developing countries, private pharmacies play an important role in providing health information and services to local communities for common health issues. The aim of this study was to ascertain medium-term impact of educational interventions on knowledge and practice of pharmacy staff regarding management of childhood diarrhea in Vietnam.

**Methods:**

This was a pre- and post-intervention study with 32 and 44 months difference from the time of the baseline survey to the conclusion of trainings and the time of the end-line survey, respectively. Interventions included in-class training for pharmacy staff, printed materials at the pharmacy, and supportive supervision. Knowledge/reported practice and actual practice of pharmacy staff were measured before and after interventions.

**Results:**

After interventions, significant improvements (p<0.01) were observed for all indexes related to pharmacy staff's knowledge about childhood diarrhea; for instance, 31% and 60% of surveyed staff asked about weight of the child and accompanying symptoms of childhood diarrhea, respectively, an increase from 11% and 45% at the baseline. Oral rehydration solution (ORS) was the most frequently reported product recommended (97% to 99%), but probiotics and antidiarrheals were the products most frequently prescribed at pharmacies. Public health facilities remained the preferred choice for referrals from pharmacies, but the use of private clinics was increasing. Consultations and advice provided to caregivers also improved, but considerable gaps between knowledge and actual practice of staff in real pharmacy settings remained.

**Conclusions:**

Educational interventions were effective in improving pharmacy staff knowledge and practice regarding management of childhood diarrhea. Knowledge and actual practice of staff in real pharmacy settings did not always correlate; there is need for a stronger regulatory and law enforcement system. Interventions to improve pharmacy practice in developing countries should be focused, comprehensive, and evidence-based.

## Introduction

Despite worldwide progress made over the past decades in reducing mortality from diarrhea, the disease (which in 2010 killed about 0.8 million children–more than the child deaths from AIDS, malaria, and measles combined) remains the second-leading killer of children aged 1 to 59 months [1]. In Vietnam, diarrhea is still considered a public health problem. A prospective study on diarrhea incidence among 1 655 children <5 years old in northern Vietnam found that the highest rates of diarrhea occurred in children <1 year old, with 3.3 cases per child per year [2]. While morbidity and mortality from childhood diarrhea have decreased, 10% of child deaths under 5 were attributed to diarrhea in 2010 [3].

In many developing countries, private pharmacies are often a patient's first point of contact with the health care system; they are a preferred source of health information, advice, and services for common health problems in local communities [4]–[6]. As pharmacy staff could potentially influence people's health-seeking behaviors and use of medicines, it is in the interest of public health researchers to study the knowledge and actual practice of staff in real pharmacy settings. Simulated clients (SC) have been used as an internationally applicable tool to assess knowledge and practice of pharmacists related to common childhood conditions, including diarrhea [7].

Research applying this method has revealed that poor history taking, inadequate information provision, poor consultations, and poor case management of childhood diarrhea were common shortcomings of pharmacists and pharmacy staff in both developed and developing countries [8]–[11]. Treatment recommended by pharmacy personnel did not align with World Health Organization (WHO) guidelines [12] for the management of diarrhea at a pharmacy. The exception is one study in Zimbabwe [13], in which the authors reported a low sale of antibiotics at private pharmacies for cases of 3.5 year old children with acute diarrhea; other studies conducted in developing countries consistently reported that antidiarrheals, probiotics, and antibiotics were the most frequently prescribed drugs by pharmacy staff for childhood diarrhea [14]–[16].

A number of studies have been conducted to examine short-term impacts of various educational interventions to improve pharmacy staff knowledge and quality of dispensing practice for cases of childhood diarrhea. Findings of one study suggest that educational intervention can produce positive impacts on staff knowledge; however, the effects it has on prescribing practice for diarrhea management were inconclusive [17]. In another study, researchers conclude that education could be an effective measure for improving drug seller prescribing practices. However, it should be noted that the sample size of this study was quite small and training methods and the gap between the intervention and surveys were unclear [18].

The objective of this paper is to report the impact of a 44-month educational intervention package on knowledge and practice of staff in pharmacy settings regarding management of childhood diarrhea from June 2008 to March 2012.

## Materials and Methods

The data for this study are derived from a project lasting over four years entitled, “Enhancing the roles of pharmacies as community health care providers,” implemented by PATH from January 2008 to June 2012. The aim of the project was to equip pharmacy staff with specific knowledge and skills related to 11 health care and pharmacy practice topics in Vietnam in order to improve the quality of services they provide. The topics include: fever, cough, diarrhea, emergency contraceptives, sexual transmitted disease, hypertension, and diabetes, rational use of antibiotics, building customer relationship, good pharmacy practice and first aid. Within the overall context of this intervention package, this paper reports on one of these eleven topics, childhood diarrhea, as this is one of the most common health problems for which people seek care at pharmacies, and one of the two simulated client scenarios (the other was emergency contraceptives) providing us with data to evaluate knowledge, reported practice, and actual practice of pharmacy staff. The project consisted of a baseline assessment (conducted in June 2008), development and implementation of interventions, and a final evaluation in March 2012 (Table 1).

**Table 1:** Study timeline.

Baseline	Training & IEC development	Trainings	Quarterly supportive supervision	End-line
June 2008	July 2008 – February 2009	April – June 2009	September 2009 – December 2011	March 2012

### Field site

Project activities were carried out in five districts, two towns, and four cities of five provinces across the country: Da Nang, Khanh Hoa, and Thua Thien Hue in the central coast; Vinh Long in the south; and Thai Nguyen in the north. The project covers a combined area of 2 095 km^2^, which is home to 40% of the population of the five provinces (2.4% of the total country population). The total population in the catchment area is 2 119 397, of whom 71.5% are urban inhabitants [19]. In terms of socio-economic indicators, these five provinces represent the upper half of the national income distribution, with a monthly average income per capita in current prices ranking 4^th^, 19^th^, 39^th^, 22^nd^, and 31^st^ respectively out of 63 provinces [20].

### Pharmacy staff survey

Using a structured questionnaire, the surveys aimed to record knowledge and reported practice of pharmacy staff on the management of childhood diarrhea. The questionnaire had two parts. The first part included general questions related to demographic data for pharmacy staff, including the highest professional education attained and average number of clients received per day. The second part consisted of specific questions on pharmacy staff knowledge and management of diarrhea, including signs of acute childhood diarrhea, signs of dehydration, danger signs requiring immediate medical care, methods for diarrhea prevention, medication recommendations, advice for caregivers, and information on referrals for severe cases of childhood diarrhea.

### Simulated client surveys

The surveys aim to assess staff attitude, actual practice, and skills in interacting with clients presenting cases of children under the age of 5 with diarrhea. A two-day intensive training was provided for all SCs. The training included role-play, rehearsing the scenario, instructions and practice on how to fill in the questionnaire, and a pilot survey in a real pharmacy setting. In addition, each SC was provided with a checklist describing actions to complete before, during, and after each visit. The following scenario and information would be provided by the SCs when entering the pharmacy: “My 14-month-old child (a son or a daughter) has diarrhea. Yesterday, s/he had 6 episodes of watery diarrhea, vomited 3 times, and looked tired. I am very worried. What can I do?” Additional information to describe the case could be revealed only if asked by pharmacy staff and included: no abdominal pain, thirsty and drank a lot of water, no fever or low-grade fever, no medication was taken, and weight of 10 kg. The SC was asked to observe the behavior and attitude of pharmacy staff, to memorize what was advised, and to buy the recommended medications. The SC was asked to debrief and record his/her observations following a standard questionnaire no later than one hour after the visit.

### Sample size

**Figure 1:**
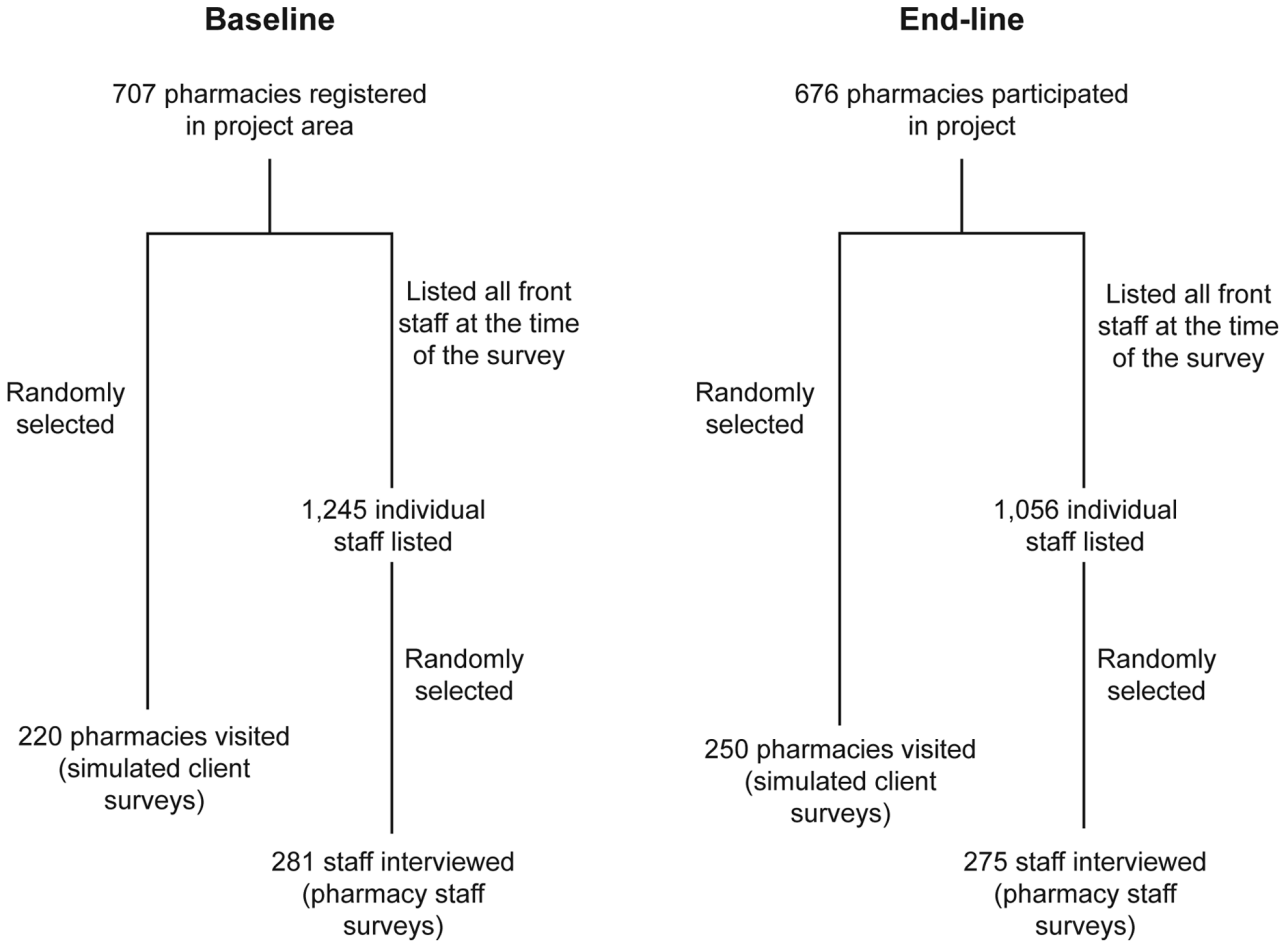
Sampling process for pharmacy staff and simulated client surveys*.

The sample size for both surveys was calculated using the WHO-recommended sample size calculation method to detect the differences in staff knowledge and practice at a level of significance (α) of 5% and power (1- β) of 80% [21]. The simple random sampling technique was employed to identify study subjects. For the pharmacy staff survey, the sampling unit was an individual pharmacy staff member; for the simulated client survey, the sampling unit was the individual pharmacy. The calculated sample size for the pharmacy staff survey was 270 with the assumption that the proportion of pharmacy staff with good knowledge on the training topics would increase by at least 25% after the intervention. A non-response rate of 4% was assumed for the baseline and 2% for the end-line surveys, giving a final planned sample size of 281 and 275 for the two surveys respectively. The same method was applied to calculate the required sample size for the simulated client surveys with a lower expectation for the level of increase in proportion of pharmacies having good practice after the intervention. The end line survey occurred during the holiday season causing a number of pharmacies expected to be temporarily out of business. A non-response rate of 12% was taken into account, giving a final sample size of 220 pharmacies for the baseline and 250 pharmacies for the end-line (Figure 1). The number of staff members and pharmacies that participated in both surveys was equally distributed across five provinces.

### Data collection

Data collection tools (questionnaires for pharmacy staff surveys and simulated client surveys) for the baseline were developed by the PATH research team in consultation with clinical experts from Bach Mai National General Hospital. All questions were designed with a closed-end format; answer options were provided but were not prompted by the interviewer. For example, the interviewees were asked what advice they would provide to a caregiver. The answers were then compared with a checklist that included options such as drink water or ORESOL as much as possible, continue breastfeeding, regularly check for signs of dehydration, and visit a physician if the situation does not improve or is getting worse. The study tools were tested at private pharmacies in two of the five provinces (Thai Nguyen and Vinh Long). Small revisions and adaptations were made in order to make these tools more appropriate with local context and language. The same questionnaires were used for the end-line evaluation.

For the pharmacy staff survey, data collectors were recruited from provincial health departments and trained by PATH researchers. For simulated client surveys, the SCs, including both men and women aged 20 to 30 with no medical or pharmacy-related background, were recruited from mass organizations (Youth Union, Women's Union, Farmer's Union). Eight and ten SCs per province participated in the baseline and end line surveys, respectively. In each province, there were two supervisors (one for the pharmacy staff survey and one for the simulated client survey) recruited to supervise the data collection process, enforce the application of standard survey procedures, and ensure the quality of data collected.

### Ethics statement

The project protocol was reviewed and approved by the provincial people's committee in each of the five project provinces, after a project appraisal process that included both technical and ethical reviews carried out by institutional review boards of the provincial department of health of Da Nang, Khanh Hoa, Thua thien Hue, Vinh Long, and Thai Nguyen in each of these provinces respectively. In the beginning of the project, an invitation letter from PATH and provincial health departments was sent to all pharmacies in the project area, and an introduction session was organized for interested pharmacies in which the project and its interventions were described and information about supportive supervision and the simulated client method was provided to all participants. At the end of the session, a written consent form was signed and submitted by each pharmacy owner willing to participate in the project. All participants were given the right to withdraw at any time without threat or disadvantage. The baseline assessment and final evaluation protocols were reviewed and approved by PATH's Research Determination Committee for non-research determination.

### Interventions

#### Training

The training materials for pharmacy staff on diarrhea management were developed based on the national guidelines for diarrhea management, integrated management of childhood illness (IMCI), issued by the Ministry of Health, and technical guidelines on management of diarrhea and communicable diseases from WHO, UNICEF, and the American Public Health Association. Trainings for pharmacy staff were conducted by experienced provincial trainers who received training of trainers provided by the project. Each training session lasted for 3.5 hours and included five parts: (i) introduction and pre-test (15 minutes); (ii) diarrhea: definition, clinical classification, causes and transmission pathways, accompanying symptoms, signs of dehydration, danger signs in need of immediate medical care, basic principles for home care (45 minutes); (iii) role of pharmacy: patient evaluation, consultation and referral, oral rehydration solutions (ORS) and instruction for use, provision of information on home-care and prevention (40 minutes); (iv) practice of diarrhea case management: role play (90 minutes); and (v) session review and post-test (20 minutes).

#### Supportive supervision

The purpose of supportive supervision visits was to reinforce the knowledge and practice of project pharmacy staff according to what they learned during training about current regulations and policy. In each project province, a supportive supervisor team was established. The supportive supervisors were health professionals recruited from the provincial health department. They were introduced to the project and provided with a three-day training that included two days of in-class training on supportive supervision and supervision skills and one day of practice at a project pharmacy. In addition, all supervisors attended the training session for pharmacy staff in order to understand expectations for staff performance in actual situations. Quarterly visits to project pharmacies were conducted by supervisors from September 2009 to December 2011 (Table 1).

#### Educational messages

Based on training materials and in consultation with national and provincial experts on internal medicine, the team developed printed materials containing key educational messages. These materials included an A4 size (hard cover – one page) Job Aid for pharmacy staff with key messages on diarrhea management as well as leaflets for distribution to clients containing information on diarrhea, home remedies (use of ORS), monitoring, and prevention. All materials were pre-tested and adjusted before mass production.

### Data entry and data analysis

The data were coded into computers using EpiData, and manually checked and screened for missing information and internal consistency. STATA 11.0 was used for data analysis. The chi-square test was employed to compare diarrhea-related knowledge and reported and actual practice of pharmacy staff on childhood diarrhea case management before and after interventions. Statistical tests used an alpha of 0.05. Data were analyzed and compared under the following groupings:

1. Case identification and patient evaluation.
2. Use of ORS and other medications.
3. Client consultation and referral.

## Results

In the baseline simulated client survey, 3 observational visits were deemed invalid due to missing and inconsistent data, leaving 217 visits for analysis; no such problems were found for the pharmacy staff survey and the two end-line surveys. A similar staff profile was found in both baseline and end-line surveys. The majority of interviewed staff members were female, with a mean age of early 40s and about 15 years of work experience. Most were intermediate and assistant pharmacists (one to two year training in secondary medical school) who assisted 46 to 62 clients per day on average (Table 2). The same staff profile pattern was found for simulated client surveys, with nearly 80% of staff attending to SCs being female, spending on average 2.6 minutes per visit at the baseline and 2.4 minutes at the end-line.

**Table 2:** Characteristics of pharmacy staffs surveyed.

	Baseline*	End-line
Gender [n (%)]		
Female	225 (80)	204 (74)
Male	56 (20)	71 (26)
Professional degree [n (%)]		
Pharmacist (post-graduate)	9 (3.2)	1 (0.4)
Pharmacist (university)	14 (5.0)	17 (6.2)
Intermediate pharmacist	155 (55.2)	179 (65.0)
Assistant pharmacist	99 (35.2)	77 (28.0)
Other	4 (1.4)	1 (0.4)
Age and experience [years]		
Average age	41.6	43.8
Average job experience	14.5	15.3
Average number of clients per day	46	62

* *Data presented for the baseline had been published in another publication* [39].

### Case identification and patient evaluation

The definition of diarrhea used in the training for pharmacy staff was *“The abrupt onset of more than 3 loose or watery stools per day”.* At the baseline, 34.2% of the interviewed staff correctly identified signs of an acute diarrhea case (“correctly identified signs” means interviewed staff member named “passing stool> = 3 times/day and loose stool” or “passing stool> = 3 times/day and watery stool” or “passing stool> = 3 times/day, loose stool, watery stool” as their answer to the question “*What are signs in a child indicating s/he has acute diarrhea?”*); and 19.9% of the respondents could name at least three signs of dehydration (dehydration signs include: thirsty, dark color/infrequent urine, dry/non-elastic skin, crying without tears, being tired/cannot drink). Similarly, 50.2% of surveyed staff knew at least three warning signs of a child with diarrhea that prompted an immediate visit to a health facility (warning signs include: fever (>39°C), blood in stool, severe vomiting, long-lasting diarrhea (>4 days), fatigue/cannot eat or drink). After intervention, all three of these indicators increased significantly to 46.6%, 44.7%, and 73.5% respectively (Table S1). In terms of actual practice, pharmacy staff asked the SCs about accompanying symptoms of diarrhea in 45.2% (n = 98) of the simulated client visits at the baseline survey, which increased to 60.4% at the end-line (p<0.01). Of those who asked these questions, most of them asked about warning signs. A similar trend was seen regarding the question on the weight of the child but, despite a big jump from the baseline, only one-third of the staff asked this question at the end-line survey (Table S2).

### Use of ORS and other medications

When asked, “what medications would you recommend for treatment of childhood diarrhea?” almost all pharmacy staff interviewed named ORS as the most frequently recommended type of product, followed by probiotics, antidiarrheals, and antibiotics. After the intervention, statistically significant improvements were found for ORS as a single choice of therapy (increased from 6.8% to 19.3%) and probiotics (decreased from 80.8% to 68.4%) (Table S1). However, results of simulated client surveys showed a different picture of actual dispensing practice. ORS was recommended at a much lower frequency–only about half of the level of self-reported practice. The most frequently recommended products in simulated client surveys were antidiarrheals at the baseline (58.1% n = 126) and probiotics at the end-line (66% n = 165). No statistically significant changes were found for the use of antidiarrheals, antibiotics, and ORS alone. Use of probiotics and the combination of ORS with other drugs increased, which contradicted reported practice (Table S2).

### Client consultation and referral

When asked “what would you do for a client who complains about a childhood diarrhea case?” in the baseline survey, the most frequent reported practice was “recommend and sell drugs,” followed by “ask for more information” and “provide consultations.” After the intervention, more staff said they would inquire about the case before making recommendations, provide consultations, and refer clients to health facilities/practitioners. Most surveyed staff knew how to prepare and use ORS, but only a few were able to list all instructions. Significant improvements were observed for each indicator about counseling home remedies and care, but only about one-fifth of the respondents were able to provide all consultation contents. Public hospitals remained the preferred option to refer severe cases, followed by commune health stations and private clinics. Increased awareness and use of the latter two types of health facilities were also observed at the end-line survey (Table S1). Results of simulated client surveys showed a similar pattern in improvements of actual pharmacy staff practice related to client consultation and referral. However, despite these improvements, less than half of the staff who attended to SCs provided such advice, and only about one-third recommended a visit to either a physician or health facility (Table S2).

## Discussion

Findings of this pre-and post-intervention study suggest that staff knowledge related to diarrhea significantly improved after interventions. Their actual practice in posing questions to clients about accompanying symptoms of diarrhea and the weight of the child to evaluate the severity of the case improved; more staff asked simulated clients questions about dehydration, warning signs, and weight of the child. However, while posing questions increased overall, still more than one-third of staff in the SC survey did not ask any of those questions. Similar to our findings, the intervention study conducted in Kenya and Indonesia found that face-to-face education can significantly increase knowledge about diarrhea and diarrhea treatment among counter attendants. The number of questions asked by attendants before making a treatment recommendation increased after intervention. However, only 24% to 38% asked about key symptoms to determine severity of diarrhea, such as blood in the stool, fever, and vomiting, and only 23% discussed signs of dehydration [22]. Although the post-intervention results of this study related to this aspect of pharmacy practice were far below our expectations, we still find them encouraging in comparison to findings of other studies using the SC method in developing countries. In a study in Sudan, 63.5% of the time staff asked no history questions [23]; in Vietnam (in 1997) only 3.4% asked about hydration status [24]; and in Pakistan, only 1.1% asked about weight of the child [25]. These results were much lower than those demonstrated in our study. Regarding referral, we found that interventions have an impact on improving the awareness and practice of staff in referring clients to a health facility or a physician, when needed. Although the number of pharmacy staff who recommended SCs to visit a physician was limited (approximately one of three after intervention), it was still substantially higher than those reported in a number of studies conducted in Vietnam and other developing countries, which demonstrated rates ranging from 3% to 17% [14], [15], [26], [27]. One factor that could partly explain the better results found in our study, and also differentiate our interventions from others, was supportive supervision. Ten quarterly supportive supervision visits were made to every intervention pharmacy between June 2009 (conclusion of trainings) and March 2012 (end-line survey).

This study reported mixed results on the management of childhood diarrhea. Nearly 100% of interviewed pharmacy staff reported ORS recommendation for a case of childhood diarrhea (both in the baseline and end-line surveys). The interventions resulted in the improved knowledge of pharmacy staff on the use of probiotics and ORS alone (which is appropriate and in line with current regulations in Vietnam), but no significant changes were observed for antidiarrheals and antibiotics. In practice, the recommendation and use of ORS significantly increased after intervention, but use of probiotics and the combination of ORS and other drugs (probiotics, antidiarrheals, antibiotics) also significantly increased. No significant changes occurred for use of antidiarrheals, antibiotics, and ORS alone, which simultaneously supports and contradicts knowledge results. Our study found big gaps between knowledge and self-reported practice of pharmacy staff, and their actual practice. This was true particularly for drug recommendations, for which probiotics and antidiarrheals were the most frequently suggested products–a finding similar to that of other studies [9], [13], [28].

Low recommendation of ORS use and irrational use of antibiotics for cases of acute childhood watery diarrhea by pharmacy personnel has been documented for decades. A study of 63 pharmacies in Sudan (in 1988) reported that ORS was recommended in only 11% of cases, while only antibiotics were dispensed in 61.9% cases of simulated clients voicing concern over her 12-month-old child's acute diarrhea [23]. In 1996, a study of 100 pharmacies in Nepal reported that 97% of retailers dispensed antimicrobials for cases of acute watery diarrhea in a child, and only 44% advised ORS therapy [26]. In Vietnam, antibiotics were the most commonly recommended medicines by pharmacy staff in similar scenarios (12-month-old child with acute diarrhea) where ORS was recommended in only one of the 29 studied pharmacies [24]. More recently, a study to assess drug seller practice for common childhood conditions in Tanzania suggested that ORS was recommended in 29% of the total 204 SC visits for watery diarrhea while antibiotics were given in 44% of the cases [16]. Our findings are consistent with that of studies in other developing countries, especially regarding discrepancies between pharmacy staff self-reported practice and their actual practice. For instance, pharmacy staff often over-reported the expected “correct practice” regarding use of ORS for childhood watery diarrhea as compared to their actual prescription practice when attending to SCs [11], [29], [30]. Overall, our results regarding actual recommendation and dispensing of ORS and antibiotics improved compared to other studies; approximately half of the visited pharmacies dispensed ORS, and the recommendation of antibiotics remained under 15% of all cases. However, this rate remained unchanged from the baseline to end-line survey, suggesting that future programs will need to focus on antibiotic use to yield better results.

Results of our study suggest that pharmacy staff performance on providing instructions to and consultations with clients could be improved with education and supportive supervision interventions even though the gap between staff knowledge and practice is still a challenge. Concerns over the quality of advice, communication, and information given at community pharmacies for common childhood illness, including diarrhea, has been raised by researchers across countries [8], [31], [32]. A number of studies have suggested that time constraints; lack of information, knowledge, and communication skills; client expectations; the motivation to maximize profit; and the negative impact of a dissatisfied customer were possible driving forces leading to poor case management and counseling at pharmacies [27], [29], [33]. The average time pharmacy staff spent per client visit found in our study (2.4 – 2.6 minutes) was substantially lower than that of a study conducted in a developed country (4.13 minutes) in which researchers concluded that the pharmacist's questioning is inadequate and needs improvement [10]. In fact, such a brief interaction in a developing country like Vietnam, where pharmacists experience a high client volume and then a limited amount of time per client visit often only allows for a few and insufficient questions to be asked by pharmacy staff before dispensing medications if at all [24]. In our study, 52% pharmacy staff (at the baseline) and 34% (at the end-line) did not pose questions before providing advices and/or medications to SCs. It was suggested that in order to translate staff knowledge into practice, to stabilize and sustain that practice, and to promote best pharmacy practice and public health, proper training and a supportive supervision system may not be enough. Law enforcement as part of a multi-component intervention to improve pharmacy practice has proved effective [34], [35] and an incentive mechanism for good pharmacy practice and increased community involvement may be required [36]. Furthermore, creating a linked referral network of trained pharmacy personnel and licensed physicians appears to be effective in encouraging timely, appropriate referrals from pharmacies to clinicians and resulted in improved health services at community level [37], [38].

This study aimed to measure both knowledge and practice of pharmacy staff on the management of childhood diarrhea before and after intervention. There was a 44-month time difference between the baseline and the end-line surveys and 32 months between the completion of trainings for pharmacy staff in all five provinces and the end-line survey. The timeframe for this study marks one of the first sets of results (if not the first) that reports on the medium-term impacts of interventions on pharmacy staff knowledge and actual practice in management of childhood diarrhea. However, study limitations need to be considered when interpreting results. First, there was no control group for both pharmacy staff and simulated client surveys, as it was part of a larger intervention project to empower community pharmacies in primary health care in Vietnam. It is assumed that some level of interaction and influence of the overall training package on pharmacy staff knowledge and practice on diarrhea management occurred, but it was not possible to measure given the scope and design of the study. Second, we were not able to match the data between the pharmacy staff and simulated client surveys. However, with substantial sample size, random sampling methods, and similar distributions of pharmacy/pharmacist characteristics, we believe our data capture a similar pattern across the two surveys. Lastly, because of staff turnover at private pharmacies and the time gap between the intervention and the end-line survey, it is possible that some surveyed staffs may not have been part of the project intervention. It must be noted, however, that pharmacies, not individual pharmacy staff, were identified as the targeted population of the project and in this study.

## Conclusion

As with other studies, this study concluded that educational interventions could be an effective tool to improve knowledge and practice of pharmacy personnel on the management of childhood diarrhea in low and middle income countries. In the pharmacy setting, knowledge and practice of staff do not always correlate as the very nature of the business could be in conflict with rational staff practice. In order to ensure that pharmacy practices are in line with national guidelines, a comprehensive, targeted intervention program should be considered and the need for a stringent law enforcement component must be thoroughly addressed. In addition, if a pharmacy is to contribute more effectively to health care, the need for an effective strategy to improve the health seeking behavior of local communities, including education for people on the perils of irrational use of medicines as a result of poor pharmacy prescription, needs to be further studied and addressed.

## Supporting Information

Table S1 Knowledge of pharmacy staff about diarrhea and management of childhood diarrhea.
(DOCX)

Table S2 Actual practice of pharmacy staff on management of childhood diarrhea.
(DOCX)
