# Correction: Improving Pharmacy Staff Knowledge and Practice on Childhood Diarrhea Management in Vietnam: Are Educational Interventions Effective?

**DOI:** 10.1371/journal.pone.0213968

**Published:** 2019-03-13

**Authors:** Minh Duc Pham, Mona Byrkit, Van Hoang Pham, Trung Pham, Thang Chien Nguyen

The first, third and fifth author’s name are spelled incorrectly. The correct names are: Minh Duc Pham, Van Hoang Pham and Thang Chien Nguyen. The correct citation is: Pham MD, Byrkit M, Pham VH, Pham T, Nguyen TC (2013) Improving Pharmacy Staff Knowledge and Practice on Childhood Diarrhea Management in Vietnam: Are Educational Interventions Effective? PLoS ONE 8(10): e74882. https://doi.org/10.1371/journal.pone.0074882
