# Supplementary material for: Improving Pharmacy Staff Knowledge and Practice on Childhood Diarrhea Management in Vietnam: Are Educational Interventions Effective?
Source: PLoS One. 2013 Oct 3;8(10):e74882. doi: 10.1371/journal.pone.0074882 (PMC3789740; doi:10.1371/journal.pone.0074882)
Supplement: Table S1 — Knowledge of pharmacy staff about diarrhea and management of childhood diarrhea. (DOCX) [file pone.0074882.s001.docx]

**Table S1. Knowledge of pharmacy staff about diarrhea and management of childhood diarrhea**

|  | *Baseline (2008) (n=281)* | | *End-line (2012) (n=275)* | | *P-value* |
| --- | --- | --- | --- | --- | --- |
|  |  | |  | |  |
|  | *No.* | *%* | *No.* | *%* |  |
| **Identification of acute childhood diarrhea and accompanying symptoms** |  |  |  |  |  |
|  |  |  |  |  |  |
| Know symptoms/signs of acute diarrhea | 96 | 34.2 | 128 | 46.6 | <0.01 |
|  |  |  |  |  |  |
| Know at least three signs of dehydration | 56 | 19.9 | 123 | 44.7 | <0.01 |
|  |  |  |  |  |  |
| Know at least three warning signs (requiring immediate medical care) | 141 | 50.2 | 202 | 73.5 | <0.01 |
|  |  |  |  |  |  |
| **Recommended medication for acute childhood diarrhea** |  |  |  |  |  |
|  |  |  |  |  |  |
| Oral rehydration solutions (ORS) | 273 | 97.2 | 271 | 98.6 | 0.12 |
|  |  |  |  |  |  |
| Probiotics | 227 | 80.8 | 188 | 68.4 | <0.01 |
|  |  |  |  |  |  |
| Antidiarrheals | 103 | 36.7 | 94 | 34.2 | 0.27 |
|  |  |  |  |  |  |
| Antibiotics | 42 | 15.0 | 34 | 12.4 | 0.19 |
|  |  |  |  |  |  |
| ORS only | 19 | 6.8 | 53 | 19.3 | <0.01 |
|  |  |  |  |  |  |
| Combination of ORS and other drugs | 245 | 87.5 | 206 | 74.9 | <0.01 |
|  |  |  |  |  |  |
| **Reported practice when receiving client with childhood diarrhea** |  |  |  |  |  |
|  |  |  |  |  |  |
| Sell drugs as prescribed | 57 | 20.3 | 48 | 17.5 | 0.2 |
|  |  |  |  |  |  |
| Ask for more information on the case | 160 | 56.9 | 188 | 68.4 | <0.01 |
|  |  |  |  |  |  |
| Recommend and sell drugs | 203 | 72.2 | 201 | 73.1 | 0.4 |
|  |  |  |  |  |  |
| Provide consultations | 136 | 48.4 | 166 | 60.4 | <0.01 |
|  |  |  |  |  |  |
| Refer client to other health facilities/practitioners | 101 | 35.9 | 118 | 42.9 | <0.05 |
|  |  |  |  |  |  |
| **Instructions and consultations for childhood diarrhea** |  |  |  |  |  |
|  |  |  |  |  |  |
| Instructions for preparation and use of ORS |  |  |  |  |  |
|  |  |  |  |  |  |
| *Mix with exact amount of boiled water as instructed* | 271 | 96.4 | 267 | 97.1 | 0.32 |
|  |  |  |  |  |  |
| *Drink as much as possible* | 114 | 40.6 | 137 | 49.8 | <0.05 |
|  |  |  |  |  |  |
| *Use within 24 hours* | 220 | 78.3 | 184 | 66.9 | <0.01 |
|  |  |  |  |  |  |
| *Do not mix with milk, soup, juices, or soft drinks* | 5 | 1.8 | 60 | 21.8 | <0.01 |
|  |  |  |  |  |  |
| *Full instructions ^b^* | 3 | 1.1 | 39 | 14.2 | <0.01 |
|  |  |  |  |  |  |
| Counseling on home remedies and care |  |  |  |  |  |
|  |  |  |  |  |  |
| *Prevent dehydration (by drinking water or ORS as much as possible)* | 225 | 80.1 | 244 | 88.7 | <0.01 |
|  |  |  |  |  |  |
| *Ensure adequate nutrition (food and breastmilk intake)* | 196 | 69.8 | 204 | 74.2 | 0.12 |
|  |  |  |  |  |  |
| *Regularly check for dehydration, warning signs* | 79 | 28.1 | 129 | 46.9 | <0.01 |
|  |  |  |  |  |  |
| *Make timely visit to medical practitioners/health facilities when needed ^c^* | 115 | 40.9 | 147 | 53.5 | <0.01 |
|  |  |  |  |  |  |
| *Full consultations ^d^* | 17 | 6.1 | 59 | 21.5 | <0.01 |
|  |  |  |  |  |  |
| **Referral (where to refer clients)** |  |  |  |  |  |
|  |  |  |  |  |  |
| Public hospital | 259 | 92.5 | 250 | 90.9 | 0.25 |
|  |  |  |  |  |  |
| Commune health station | 74 | 26.4 | 155 | 56.4 | <0.01 |
|  |  |  |  |  |  |
| Private clinics | 42 | 15.0 | 93 | 33.8 | <0.01 |

^a^ *Named ORS as the only remedy for childhood diarrhea (not any other drugs)*

*^b^ Named all four instructions as mentioned above*

^c “^*“When needed” means having dehydration or warning signs, and the child’s condition is not improving or is getting worse*

*^d^ Named all four consultations as mentioned above*
