# Supplementary material for: Improving Pharmacy Staff Knowledge and Practice on Childhood Diarrhea Management in Vietnam: Are Educational Interventions Effective?
Source: PLoS One. 2013 Oct 3;8(10):e74882. doi: 10.1371/journal.pone.0074882 (PMC3789740; doi:10.1371/journal.pone.0074882)
Supplement: Table S2 — Actual practice of pharmacy staff on management of childhood diarrhea. (DOCX) [file pone.0074882.s002.docx]

**Table S2: Actual practice of pharmacy staff on management of childhood diarrhea**

|  | Baseline (2008) | | End-line (2012) | | P-value |
| --- | --- | --- | --- | --- | --- |
|  |  | |  | |  |
|  | *No.* | *%* | *No.* | *%* |  |
|  |  |  |  |  |  |
| **Enquiries about the case** |  |  |  |  |  |
|  |  |  |  |  |  |
| Asked about accompanying symptoms/signs of a childhood diarrhea | 98 | 45.2 | 151 | 60.4 | <0.01 |
|  |  |  |  |  |  |
| *Dehydration signs* | *37* | *37.8* | *63* | *41.7* | *0.26* |
|  |  |  |  |  |  |
| *Warning signs (required immediate medical care)* | *85* | *86.7* | *130* | *86.1* | *0.44* |
|  |  |  |  |  |  |
| Asked about weight of the child | 23 | 10.6 | 78 | 31.2 | <0.01 |
|  |  |  |  |  |  |
| None of the above questions asked | 112 | 51.6 | 86 | 34.4 | <0.01 |
|  |  |  |  |  |  |
| **Medication dispensing practice** |  |  |  |  |  |
|  |  |  |  |  |  |
| No drug dispensed and patient referred to health facilities or practitioners | 13 | 6.0 | 11 | 4.4 | 0.22 |
|  |  |  |  |  |  |
| Oral rehydration solutions (ORS) | 91 | 41.9 | 131 | 52.4 | <0.05 |
|  |  |  |  |  |  |
| Probiotics | 111 | 51.2 | 165 | 66.0 | <0.01 |
|  |  |  |  |  |  |
| Antidiarrheals | 126 | 58.1 | 130 | 52.0 | 0.09 |
|  |  |  |  |  |  |
| Antibiotics | 32 | 14.8 | 35 | 14.0 | 0.4 |
|  |  |  |  |  |  |
| ORS only ^a^ | 13 | 6.0 | 12 | 4.8 | 0.28 |
|  |  |  |  |  |  |
| Combination of ORS and other drugs | 77 | 35.5 | 117 | 46.8 | <0.01 |
|  |  |  |  |  |  |
| **Information and consultations given** |  |  |  |  |  |
|  |  |  |  |  |  |
| Provide instructions for preparation and use of ORS | 68 | 31.3 | 105 | 42.0 | <0.01 |
|  |  |  |  |  |  |
| *Drink as much as possible* | *26* | *38.2* | *72* | *68.6* | *<0.01* |
|  |  |  |  |  |  |
| *Mix with exact amount of boiled water as instructed* | *44* | *64.7* | *81* | *77.1* | *<0.05* |
|  |  |  |  |  |  |
| *Do not mix with milk, soup, juices, or soft drinks* | *3* | *4.4* | *11* | *10.5* | *0.07* |
|  |  |  |  |  |  |
| *Use within 24 hours* | *18* | *26.5* | *63* | *60.0* | *<0.01* |
|  |  |  |  |  |  |
| *Full instructions ^b^* | *0* | *0* | *2* | *1.9* | *0.12* |
|  |  |  |  |  |  |
| Prevent dehydration (by drinking water or ORS as much as possible) | 22 | 10.1 | 73 | 29.2 | <0.01 |
|  |  |  |  |  |  |
| Ensure adequate nutrition (food and breastmilk intake) | 20 | 9.2 | 96 | 38.4 | <0.01 |
|  |  |  |  |  |  |
| Regularly check for dehydration or warning signs | 9 | 4.2 | 29 | 11.6 | <0.01 |
|  |  |  |  |  |  |
| Make timely visit to medical practitioners/health facilities when needed^c^ | 34 | 15.7 | 64 | 25.6 | <0.01 |
|  |  |  |  |  |  |
| Full consultations ^d^ | 0 | 0 | 6 | 2.4 | <0.05 |
|  |  |  |  |  |  |
| Provide consultation on dehydration signs | 27 | 12.4 | 57 | 22.8 | <0.01 |
|  |  |  |  |  |  |
| Provide consultation on warning signs | 49 | 22.6 | 94 | 37.6 | <0.01 |
|  |  |  |  |  |  |
| Provide consultation on methods for prevention | 3 | 1.4 | 37 | 14.8 | <0.01 |
|  |  |  |  |  |  |
| **Referral practice** |  |  |  |  |  |
|  |  |  |  |  |  |
| Refer patient to other health facilities/practitioners | 53 | 24.4 | 88 | 35.2 | <0.01 |
|  |  |  |  |  |  |
| *Public hospital* | *39* | *73.6* | *73* | *83.0* | *0.09* |
|  |  |  |  |  |  |
| *Commune health station* | *7* | *13.2* | *24* | *27.3* | <0.05 |
|  |  |  |  |  |  |
| *Private clinic/practitioners* | *2* | *3.8* | *13* | *11.4* | *0.05* |
|  |  |  |  |  |  |
| *Others (nearest health facilities/practitioners, other pharmacies)* | *8* | *15.0* | *7* | *7.9* | *0.09* |

^a^ *Dispensed ORS as the only remedy during SC visits*

*^b^ Provided all four instructions as mentioned above*

^c^ *“As needed” means having dehydration or warning signs, and the child’s condition is not improving or is getting worse*

*^d^ Provided all four consultations as mentioned above*
